# Two generalized Lyapunov-type inequalities for a fractional *p*-Laplacian equation with fractional boundary conditions

**DOI:** 10.1186/s13660-017-1374-3

**Published:** 2017-05-03

**Authors:** Yang Liu, Dapeng Xie, Dandan Yang, Chuanzhi Bai

**Affiliations:** 1 Department of Mathematics, Hefei Normal University, Hefei, Anhui 230061 P.R. China; 2 Department of Mathematics, Huaiyin Normal University, Huaian, Jiangsu 223300 P.R. China

**Keywords:** 26A33, 34A08, 76F70, fractional boundary value problem, Lyapunov-type inequality, *p*-Laplacian operator, Guo-Krasnoselskii fixed point theorem

## Abstract

In this paper, we investigate the existence of positive solutions for the boundary value problem of nonlinear fractional differential equation with mixed fractional derivatives and *p*-Laplacian operator. Then we establish two smart generalizations of Lyapunov-type inequalities. Some applications are given to demonstrate the effectiveness of the new results.

## Introduction

Lyapunov’s inequality [1] has proved to be very useful in various problems related with differential equations; for examples, see [2, 3] and the references therein. Recently, many researchers have given some Lyapunov-type inequalities for different classes of fractional boundary value problems (see [4–10]). In [7], Ferreira investigated a Lyapunov-type inequality for the fractional boundary value problem

1.1 $$ \textstyle\begin{cases} D_{a^{+}}^{\alpha} y(t) + q(t)y(t) = 0,\quad a < t < b, \\ y(a)=y(b)=0, \end{cases} $$

where $D_{a^{+}}^{\alpha}$ is the Riemann-Liouville fractional derivative of order *α*, $1 < \alpha\le2$, *a* and *b* are consecutive zeros, and *q* is a real and continuous function. It was proved that if (1.1) has a nontrivial solution, then

1.2 $$ \int_{a}^{b} \bigl\vert q(t) \bigr\vert \, \mathrm{d}s > \Gamma(\alpha) \biggl(\frac{4}{b-a} \biggr)^{\alpha-1}. $$

Obviously, if we set $\alpha= 2$ in (1.2), one can obtain the classical Lyapunov inequality [1].

In [8], Jleli and Samet considered the fractional differential equation

1.3 $$ {}^{C} D_{a^{+}}^{\alpha} y(t) + q(t)y(t) = 0,\quad a < t < b, 1 < \alpha\le2, $$

with the mixed boundary conditions

1.4 $$ y(a)=y'(b)=0 $$

or

1.5 $$ y'(a)=y(b)=0, $$

where $^{C} D_{a^{+}}^{\alpha}$ is the Caputo fractional derivative of order $1 < \alpha\le2$. For boundary conditions (1.4) and (1.5), two Lyapunov-type inequalities were established, respectively, as follows:

1.6 $$ \int_{a}^{b} (b-s)^{\alpha-2} \bigl\vert q(s) \bigr\vert \,\mathrm{d}s \geq\frac{\Gamma(\alpha)}{\operatorname{max}\{\alpha-1 , 2-\alpha\}(b-a)} $$

and

1.7 $$ \int_{a}^{b} (b-s)^{\alpha-1} \bigl\vert q(s) \bigr\vert \,\mathrm{d}s \ge\Gamma(\alpha). $$

Recently, we considered in [11] the same equation (1.3) with the fractional boundary condition

$$ y(a) = {}^{C} D_{a^{+}}^{\beta} y(b)= 0, $$

where $0 < \beta\le1$.

In [12], Arifi *et al.* considered the following nonlinear fractional boundary value problem with *p*-Laplacian operator:

1.8 $$ \textstyle\begin{cases} D_{a^{+}}^{\beta} (\Phi_{p}(D_{a^{+}}^{\alpha} u(t)) ) + \chi(t) \Phi_{p}(u(t))=0,\quad a < t < b, \\ u(a)= u^{\prime}(a)=u^{\prime}(b)=0,\qquad D_{a^{+}}^{\alpha} u(a) = D_{a^{+}}^{\alpha} u(b) = 0, \end{cases} $$

where $2 < \alpha\le3$, $1 < \beta\le2$, $D_{a^{+}}^{\alpha}$, $D_{a^{+}}^{\beta}$ are the Riemann-Liouville fractional derivative of orders *α*, *β*, $\Phi_{p}(s) = \vert s \vert ^{p-2}s$, $p > 1$, and $\chi: [a, b] \to \mathbb{R}$ is a continuous function. It was proved that if (1.8) has a nontrivial continuous solution, then

1.9 $$\begin{aligned} &\int_{a}^{b} (b-s)^{\beta-1} (s-a)^{\beta-1} \bigl\vert \chi(s) \bigr\vert \,\mathrm{d}s \\ &\quad\ge \bigl( \Gamma(\alpha) \bigr)^{p-1} \Gamma(\beta) (b-a)^{\beta-1} \biggl( \int_{a}^{b} (b-s)^{\alpha-2} (s-a) \,\mathrm{d}s \biggr)^{1-p}. \end{aligned}$$

More recently, Chidouh and Torres in [13] considered the following boundary value problem:

1.10 $$ \textstyle\begin{cases} D_{a^{+}}^{\alpha} y(t) + q(t) f(y(t)) = 0,\quad a < t < b, \\ y(a)=y(b)=0, \end{cases} $$

where $D_{a^{+}}^{\alpha}$ is the Riemann-Liouville fractional derivative with $1 < \alpha\le2$, and $q: [a, b] \to\mathbb{R}_{+}$ is a nontrivial Lebesgue integrable function. Under the assumption that the nonlinear term $f \in C(\mathbb{R}_{+}, \mathbb{R}_{+})$ is a concave and decreasing function, it was proved that if (1.10) has a nontrivial solution, then

1.11 $$ \int_{a}^{b} \bigl\vert q(t) \bigr\vert \, \mathrm{d}s > \frac{4^{\alpha-1} \Gamma(\alpha) \eta}{(b-a)^{\alpha-1} f(\eta)}, $$

where $\eta= \max_{t \in[a, b]} y(t)$. Obviously, if we set $f(y) = y$ in (1.11), one can obtain a Lyapunov inequality (1.2).

Motivated by the above work, in this paper, we consider the fractional boundary value problem

1.12 $$ \textstyle\begin{cases} D_{a^{+}}^{\beta} (\Phi_{p}(^{C} D_{a^{+}}^{\alpha} u(t)) ) - k(t) f(u(t)) = 0,\quad a < t < b, \\ u^{\prime}(a)= {} ^{C} D_{a^{+}}^{\alpha} u(a) = 0,\qquad u(b) = ^{C} D_{a^{+}}^{\alpha} u(b) = 0, \end{cases} $$

where $1 < \alpha, \beta\leq2$, and $k:[a , b]\rightarrow R$ is a continuous function. We write (1.12) as an equivalent integral equation and then, by using some properties of its Green function and the Guo-Krasnoselskii fixed point theorem, we can obtain our first result asserting existence of nontrivial positive solutions to problem (1.12). Then, under some assumptions on the nonlinear term *f*, we are able to get two corresponding Lyapunov-type inequalities. Finally in this paper, two corollaries and an example are given to demonstrate the effectiveness of the obtained results.

## Preliminaries

In this section, we recall the definitions of the Riemann-Liouville fractional integral, fractional derivative, and the Caputo fractional derivative and give some lemmas which are useful in this article. For more details, we refer to [14, 15].

### Definition 2.1

Let $\alpha\geq0$ and *f* be a real function defined on $[a, b]$. The Riemann-Liouville fractional integral of order *α* is defined by $_{a} I^{0} f \equiv f$ and

$$\bigl(I_{a^{+}}^{\alpha} f \bigr) (t)=\frac{1}{\Gamma(\alpha)} \int_{a}^{t} (t-s)^{\alpha-1}f(s) \,\mathrm{d}s,\quad \alpha>0, t \in[a, b]. $$

### Definition 2.2

The Riemann-Liouville fractional derivative of order $\alpha> 0$ of a function $f: [a, b] \to\mathbb{R}$ is given by

$$\bigl(D_{a^{+}}^{\alpha} f \bigr) (t) = \frac{1}{\Gamma(n-\alpha)} \frac{d^{n}}{dt^{n}} \int_{a}^{t} \frac{f(s)}{(t-s)^{\alpha-n+1}} \,\mathrm{d}s, $$

where *n* is the smallest integer greater or equal to *α* and Γ denotes the Gamma function.

### Definition 2.3

The Caputo derivative of fractional order $\alpha\ge0$ is defined by $^{C} D_{a^{+}}^{0} f \equiv f$ and

$$\bigl(^{C} D_{a^{+}}^{\alpha} f \bigr) (t) = \frac{1}{\Gamma(n-\alpha)} \int_{a}^{t} (t-s)^{n-\alpha-1} f^{(n)}(s) \,\mathrm{d}s,\quad \alpha> 0, t \in[a, b], $$

where *n* is the smallest integer greater or equal to *α*.

### Lemma 2.1

Guo-Krasnoselskii fixed point theorem [16]

*Let* *X* *be a Banach space and let* $P \subset X$ *be a cone*. *Assume* $\Omega_{1}$ *and* $\Omega_{2}$ *are bounded open subsets of* *X* *with* $0 \in\Omega_{1} \subset\bar{\Omega}_{1} \subset\Omega_{2}$, *and let* $T: P \cap(\bar{\Omega}_{2} \setminus\Omega _{1}) \to P$ *be a completely continuous operator such that*

(i) $\Vert Tu \Vert \ge \Vert u \Vert $ *for any* $u \in P \cap\partial\Omega_{1}$ *and* $\Vert Tu \Vert \le \Vert u \Vert $ *for any* $u \in P \cap\partial\Omega_{2}$; *or*
(ii) $\Vert Tu \Vert \le \Vert u \Vert $ *for any* $u \in P \cap\partial\Omega_{1}$ *and* $\Vert Tu \Vert \ge \Vert u \Vert $ *for any* $u \in P \cap\partial\Omega_{2}$.

*Then* *T* *has a fixed point in* $P \cap(\bar{\Omega}_{2} \setminus\Omega _{1}) $.

### Lemma 2.2

Jensen’s inequality [17]

*Let* *ν* *be a positive measure and let* Ω *be a measurable set with* $\nu(\Omega)=1$. *Let* *I* *be an interval and suppose that* *u* *is a real function in* $L(d \nu)$ *with* $u(t) \in I$ *for all* $t \in\Omega$. *If* *f* *is convex on* *I*, *then*

2.1 $$ f \biggl( \int_{\Omega} u(t) \,\mathrm{d} \nu(t) \biggr) \le \int_{\Omega} (f \circ u) (t) \,\mathrm{d} \nu(t). $$

*If* *f* *is concave on* *I*, *then the inequality* (2.1) *holds with* ≤ *substituted by* ≥.

## Main results

We begin to write problem (1.12) in its equivalent integral form.

### Lemma 3.1

*If* $u \in C[a, b]$, $1 < \alpha, \beta\leq2$, $p > 1$, *and* $\frac{1}{p} + \frac{1}{q} = 1$. *Then BVP* (1.12) *has a unique solution*

3.1 $$ u(t) = \int_{a}^{b} G(t,s) \Phi_{q} \biggl( \int_{a}^{b} H(s, \tau) k(\tau)f \bigl(u(\tau) \bigr) \,\mathrm{d}\tau \biggr) \,\mathrm{d}s, $$

*where*

3.2 $$ G(t,s) = \frac{1}{\Gamma(\alpha)} \textstyle\begin{cases} (b-s)^{\alpha-1} - (t-s)^{\alpha-1}, & a\leq s\leq t\leq b, \\ (b-s)^{\alpha-1}, & a \leq t \le s \leq b, \end{cases} $$

*and*

3.3 $$ H(s,\tau) = \frac{1}{\Gamma(\beta)} \textstyle\begin{cases} (\frac{s-a}{b-a} )^{\beta-1}(b-\tau)^{\beta-1} - (s-\tau)^{\beta-1}, & a \leq\tau\leq s \le b, \\ (\frac{s-a}{b-a} )^{\beta-1}(b-\tau)^{\beta-1}, & a \leq s \le\tau \le b. \end{cases} $$

### Proof

Set $\Phi_{p}(^{c} D_{a^{+}}^{\alpha} u(t))=v(t) $. Then BVP (1.12) can be turned into the following coupled boundary value problems:

3.4 $$ \textstyle\begin{cases} D_{a^{+}}^{\beta} v(t) = k(t) f(u(t)),\quad a < t < b, \\ v(a) = v(b) = 0, \end{cases} $$

and

3.5 $$ \textstyle\begin{cases} {}^{c} D_{a^{+}}^{\alpha} u(t) = \Phi_{q}(v(t)),\quad a < t < b, \\ u^{\prime}(a) = u(b) = 0. \end{cases} $$

From Lemma 2 of [7], we see that BVP (3.4) has a unique solution, which is given by

3.6 $$ v(t) = - \int_{a}^{b} H(t, s) k(s) f \bigl(u(s) \bigr) \, \mathrm{d}s, $$

where $H(t, s)$ is as in (3.3). Moreover, by Lemma 5 of [8], we see that BVP (3.5) has a unique solution, which is given by

3.7 $$ u(t) = - \int_{a}^{b} G(t, s) \Phi_{q} \bigl(v(s) \bigr) \,\mathrm{d}s, $$

where $G(t, s)$ is as in (3.2). Substitute (3.6) into (3.7), we see that BVP (1.12) has a unique solution which is given by (3.1). □

### Lemma 3.2

*The Green’s function* *H* *defined by* (3.3) *satisfies the following properties*:

1. $H(t, s) \ge0$ *for all* $a \le t, s \le b$;
2. $\max_{t \in[a, b]} H(t, s) = H(s, s), s \in[a, b]$;
3. $H(s, s)$ *has a unique maximum given by*
  $$\max_{s \in[a, b]} H(s, s) = \frac{(b-a)^{\beta-1}}{4^{\beta-1} \Gamma (\beta)}; $$
4. ${ \min_{t \in [\frac{3a+b}{4}, \frac{a+3b}{4} ]} H(t, s) \ge \sigma(s) H(s, s), a < s < b,}$

*where*

3.8 $$\begin{aligned} \begin{aligned} &\sigma(s) = \textstyle\begin{cases} \frac{ (\frac{3(b-a)(b-s)}{4} )^{\beta-1} - (b-a)^{\beta-1} (\frac {a+3b}{4}-s )^{\beta-1}}{(s-a)^{\beta-1} (b-s)^{\beta-1}} & \textit{if } s \in (a, c_{\beta} ], \\ (\frac{b-a}{4(s-a)} )^{\beta-1} & \textit{if } s \in [c_{\beta}, b ), \end{cases}\displaystyle \\ &c_{\beta}:= \frac{\frac{a+3b}{4} - b A_{\beta}}{1-A_{\beta}},\qquad A_{\beta } = \biggl( \biggl( \frac{3}{4} \biggr)^{\beta-1} - \biggl(\frac{1}{4} \biggr)^{\beta-1} \biggr)^{\frac{1}{\beta-1}}. \end{aligned} \end{aligned}$$

### Proof

The first three properties are proved in [7]. For convenience, we set

$$h_{1}(t, s) = \frac{1}{\Gamma(\beta)} \biggl( \biggl(\frac{t-a}{b-a} \biggr)^{\beta-1}(b-s)^{\beta-1} - (t-s)^{\beta-1} \biggr),\quad s \le t $$

and

$$h_{2}(t, s) = \frac{1}{\Gamma(\beta)} \biggl(\frac{t-a}{b-a} \biggr)^{\beta-1}(b-s)^{\beta-1},\quad t \le s. $$

From [7], we know that $h_{1}(t, s)$ is decreasing with respect to *t* for $s \le t$, and $h_{2}(t, s)$ is increasing with respect to *t* for $t \le s$. Thus

$$\min_{t \in [\frac{3a+b}{4}, \frac{a+3b}{4} ]} H(t, s) = \textstyle\begin{cases} h_{1} (\frac{a+3b}{4}, s ) & \text{if } s \in (a, \frac{3a+b}{4} ], \\ \min \{h_{1} (\frac{a+3b}{4}, s ), h_{2} (\frac{3a+b}{4}, s ) \} & \text{if } s \in [\frac{3a+b}{4}, \frac{a+3b}{4} ], \\ h_{2} (\frac{3a+b}{4}, s ) & \text{if } s \in [\frac{a+3b}{4}, b ). \end{cases} $$

From

$$h_{1} \biggl(\frac{a+3b}{4}, s \biggr) = h_{2} \biggl( \frac{3a+b}{4}, s \biggr) $$

we have

$$\biggl(\frac{\frac{a+3b}{4}-s}{b-s} \biggr)^{\beta-1} = \biggl(\frac {3}{4} \biggr)^{\beta-1} - \biggl(\frac{1}{4} \biggr)^{\beta-1}, $$

which implies that

$$s = \frac{\frac{a+3b}{4} - b A_{\beta}}{1-A_{\beta}} = c_{\beta}, $$

where $c_{\beta}$ and $A_{\beta}$ are as in (3.8). It is easy to check that $A_{\beta} < \frac{3}{4}$ and $c_{\beta} < \frac{a+3b}{4}$. On the other hand, since

$$3^{\beta-1} + 8^{\beta-1} \ge2 \sqrt{3^{\beta-1} 8^{\beta-1}} \ge4^{\frac{\beta-1}{2}} 3^{\frac{\beta-1}{2}} 8^{\frac {\beta-1}{2}} = 96^{\frac{\beta-1}{2}} > 9^{\beta-1}, $$

we have

$$\biggl(\frac{3}{4} \biggr)^{\beta-1} < \biggl(\frac{2}{3} \biggr)^{\beta-1} + \biggl(\frac{1}{4} \biggr)^{\beta-1}, $$

from which we deduce that $A_{\beta} < \frac{2}{3}$ and $c_{\beta} > \frac{3a+b}{4}$. So $c_{\beta} \in (\frac{3a+b}{4}, \frac{a+3b}{4} )$ is the unique solution of the equation $h_{1} (\frac {a+3b}{4}, s ) = h_{2} (\frac{3a+b}{4}, s )$. Hence

$$\begin{aligned} \min_{t \in [\frac{3a+b}{4}, \frac{a+3b}{4} ]} H(t, s) = {}& \textstyle\begin{cases} h_{1} (\frac{a+3b}{4}, s ) & \text{if } s \in (a, c_{\beta} ], \\ h_{2} (\frac{3a+b}{4}, s ) & \text{if } s \in [c_{\beta}, b ) \end{cases}\displaystyle \\={}& \frac{1}{\Gamma(\beta)} \textstyle\begin{cases} (\frac{3(b-s)}{4} )^{\beta-1} - (\frac{a+3b}{4} - s )^{\beta-1} & \text{if } s \in (a, c_{\beta} ], \\ (\frac{b-s}{4} )^{\beta-1} & \text{if } s \in [c_{\beta}, b ) \end{cases}\displaystyle \\ \ge{}&\sigma(s) H(s, s). \end{aligned}$$

 □

### Remark 3.1

Since $\frac{2a+b}{3} < \frac{2b-a}{3}$ implies $3a < b$, we see that the conclusion of Lemma 7(4) in [13] only holds for $a < \frac{b}{3}$.

### Lemma 3.3

[8]

*The Green’s function* *G* *defined by* (3.2) *satisfies the following properties*:

(i) $0 \le G(t, s) \le G(s, s) = \frac{1}{\Gamma(\alpha)}(b-s)^{\alpha -1} $ *for all* $a \le t, s \le b$;
(ii) $G(s, s)$ *has a unique maximum given by*
  $$\max_{s \in[a, b]} G(s, s) = \frac{1}{\Gamma(\alpha)}(b-a)^{\alpha-1}; $$
(iii) ${ \min_{t \in [\frac{3a+b}{4}, \frac{a+3b}{4} ]} G(t, s) \ge \mu(s) G(s, s), a < s < b,}$ *where*
  $$\mu(s) = \textstyle\begin{cases} 1- (\frac{\frac{a+3b}{4}-s}{b-s} )^{\alpha-1} & \textit{if } s \in (a, \frac{a+3b}{4} ], \\ 1 & \textit{if } s \in [\frac{a+3b}{4}, b ). \end{cases} $$

*Let* $E = C[a, b]$ *be endowed with the norm* $\Vert x \Vert = \max_{t \in[a, b]} \vert x(t) \vert $. *Define the cone* $P \subset E$ *by*

$$P = \bigl\{ x \in E| x(t) \ge0 \ \forall t \in[a, b] \textit{ and } \Vert x \Vert \neq0 \bigr\} . $$

### Theorem 3.4

*Let* $k: [a, b] \to\mathbb{R}_{+} = [0, + \infty)$ *be a nontrivial Lebesgue integrable function*. *Suppose that there exist two positive constants* $r_{2} > r_{1} > 0$ *such that the following assumptions*:

1. $f(x) \ge\rho\Phi_{p}(r_{1})$ *for* $x \in[0, r_{1}]$,
2. $f(x) \le\omega\Phi_{p}(r_{2})$ *for* $x \in[0, r_{2}]$,

*are satisfied*, *where*

$$\rho= \biggl[ \int_{a}^{b} \sigma(\tau) H(\tau, \tau) k(\tau) \, \mathrm{d}\tau\times\Phi_{p} \biggl( \int_{\frac{3a+b}{4}}^{\frac{a+3b}{4}} \mu(s) G(s, s) \,\mathrm{d}s \biggr) \biggr]^{-1} $$

*and*

$$\omega= \biggl[ \int_{a}^{b} H(\tau, \tau) k(\tau) \,\mathrm{d}\tau \times\Phi_{p} \biggl( \int_{a}^{b} G(s,s) \,\mathrm{d}s \biggr) \biggr]^{-1}. $$

*Then FBVP* (1.12) *has at least one nontrivial positive solution* *u* *belonging to* *E* *such that* $r_{1} \le \Vert u \Vert \le r_{2}$.

### Proof

Let $T: P \to E$ be the operator defined by

$$Tu(t) = \int_{a}^{b} G(t,s) \Phi_{q} \biggl( \int_{a}^{b} H(s, \tau) k(\tau)f\bigl(u(\tau)\bigr) \,\mathrm{d}\tau \biggr) \,\mathrm{d}s. $$

By using the Arzela-Ascoli theorem, we can prove that $T: P \to P$ is completely continuous. Let $\Omega_{i} = \{u \in P: \Vert u \Vert \le r_{i}\}$, $i = 1, 2$. From (H1), and Lemmas 3.2 and 3.3, we obtain for $t \in [\frac{3a+b}{4}, \frac{a+3b}{4} ]$ and $u \in P \cap \partial\Omega_{1}$

$$\begin{aligned} (Tu) (t) &\ge \int_{a}^{b} \min_{t \in [\frac{3a+b}{4}, \frac{a+3b}{4} ]} G(t,s) \Phi_{q} \biggl( \int_{a}^{b} H(s, \tau) k(\tau)f \bigl(u(\tau) \bigr) \,\mathrm{d} \tau \biggr) \,\mathrm{d}s \\ &\ge \int_{a}^{b} \mu(s) G(s, s) \Phi_{q} \biggl( \int_{a}^{b} H(s, \tau) k(\tau)f \bigl(u(\tau) \bigr) \,\mathrm{d} \tau \biggr) \,\mathrm{d}s \\ &\ge \int_{\frac{3a+b}{4}}^{\frac{a+3b}{4}} \mu(s) G(s, s) \,\mathrm{d}s \cdot \Phi_{q} \biggl( \int_{a}^{b} \min_{s \in [\frac{3a+b}{4}, \frac{a+3b}{4} ]} H(s, \tau) k(\tau)f \bigl(u(\tau) \bigr) \,\mathrm{d}\tau \biggr) \\ &\ge \int_{\frac{3a+b}{4}}^{\frac{a+3b}{4}} \mu(s) G(s, s) \,\mathrm{d}s \cdot \Phi_{q} \biggl( \int_{a}^{b} \sigma(\tau) H(\tau, \tau) k(\tau)f \bigl(u(\tau) \bigr) \,\mathrm{d}\tau \biggr) \\ &\ge \int_{\frac{3a+b}{4}}^{\frac{a+3b}{4}} \mu(s) G(s, s) \,\mathrm{d}s \cdot \Phi_{q} \biggl( \int_{a}^{b} \sigma(\tau) H(\tau, \tau) k(\tau) \, \mathrm{d}\tau \biggr) \Phi_{q}(\rho) \cdot r_{1} \\ &= \Vert u \Vert . \end{aligned}$$

Hence, $\Vert Tu \Vert \ge \Vert u \Vert $ for $u \in P \cap\partial\Omega_{1}$. On the other hand, from (H2), Lemmas 3.2 and 3.3, we have

$$\begin{aligned} \Vert Tu \Vert &= \max_{t \in[a, b]} \int_{a}^{b} G(t,s) \Phi_{q} \biggl( \int_{a}^{b} H(s, \tau) k(\tau)f \bigl(u(\tau) \bigr) \,\mathrm{d} \tau \biggr) \,\mathrm{d}s \\ &\le \int_{a}^{b} G(s,s) \,\mathrm{d}s \cdot \Phi_{q} \biggl( \int_{a}^{b} H(\tau, \tau) k(\tau)f \bigl(u(\tau) \bigr) \,\mathrm{d}\tau \biggr) \\ &\le \int_{a}^{b} G(s,s) \,\mathrm{d}s \cdot \Phi_{q} \biggl( \int_{a}^{b} H(\tau, \tau) k(\tau) \,\mathrm{d}\tau \biggr) \Phi_{q}(\omega) r_{2} = \Vert u \Vert \end{aligned}$$

for $u \in P \cap\partial\Omega_{2}$. Thus, by Lemma 2.1, we see that the operator *T* has a fixed point in $u \in P \cap(\bar{\Omega}_{2} \setminus\Omega_{1})$ with $r_{1} \le \Vert u \Vert \le r_{2}$, and clearly *u* is a positive solution for FBVP (1.12).

Next, we will give two Lyapunov inequalities for FBVP (1.12). □

### Theorem 3.5

*Let* $k: [a, b] \to\mathbb{R}_{+}$ *be a real nontrivial Lebesgue function*. *Suppose that there exists a positive constant* *M* *satisfying* $0 \le f(x) \le M \Phi_{p}(x)$ *for any* $x \in\mathbb{R}_{+}$. *If* (1.12) *has a nontrivial solution in* *P*, *then the following Lyapunov inequality holds*:

$$\int_{a}^{b} k(\tau) \,\mathrm{d}\tau> \frac{4^{\beta-1} \Gamma(\beta)}{M (b-a)^{\beta-1}} \Phi_{p} \biggl(\frac {\Gamma(\alpha+1)}{(b-a)^{\alpha}} \biggr). $$

### Proof

Assume $u \in P$ is a nontrivial solution for (1.12), then $\Vert u \Vert \neq0$. From (3.1), and Lemmas 3.2 and 3.3, $\forall t \in[a, b]$, we have

$$\begin{aligned} 0 &\le u(t) \le \int_{a}^{b} G(s,s) \Phi_{q} \biggl( \int_{a}^{b} H(\tau, \tau) k(\tau)f \bigl(u(\tau) \bigr) \,\mathrm{d}\tau \biggr) \,\mathrm{d}s \\ &< \int_{a}^{b} G(s,s) \,\mathrm{d}s \cdot \Phi_{q} \biggl( \int_{a}^{b} H(\tau, \tau) k(\tau) \,\mathrm{d}\tau \biggr) \Phi_{q}(M) \Vert u \Vert \\ &\le\frac{1}{\Gamma(\alpha)} \int_{a}^{b} (b-s)^{\alpha-1} \,\mathrm{d}s \cdot \Phi_{q} \biggl( \int_{a}^{b} \frac{(b-a)^{\beta-1}}{4^{\beta-1} \Gamma(\beta)} k(\tau) \,\mathrm{d} \tau \biggr) \Phi_{q}(M) \Vert u \Vert \\ &= \frac{1}{\Gamma(\alpha+1)} (b-a)^{\alpha} \cdot\Phi_{q} \biggl( \frac{(b-a)^{\beta-1}}{4^{\beta-1} \Gamma(\beta)} \biggr) \Phi_{q} \biggl( \int_{a}^{b} k(\tau) \,\mathrm{d}\tau \biggr) \Phi_{q}(M) \Vert u \Vert , \end{aligned}$$

which implies that

$$\int_{a}^{b} k(\tau) \,\mathrm{d}\tau> \frac{4^{\beta-1} \Gamma(\beta)}{M (b-a)^{\beta-1}} \Phi_{p} \biggl(\frac {\Gamma(\alpha+1)}{(b-a)^{\alpha}} \biggr). $$

 □

### Theorem 3.6

*Let* $k: [a, b] \to\mathbb{R}_{+}$ *be a real nontrivial Lebesgue function*. *Assume that* $f \in C(\mathbb{R}_{+}, \mathbb{R}_{+})$ *is a concave and nondecreasing function*. *If* (1.12) *has a nontrivial solution* $u \in P$, *then*

$$\int_{a}^{b} k(\tau) \,\mathrm{d}\tau> \frac{4^{\beta-1} \Gamma(\beta) \Phi_{p}(\Gamma(\alpha+1)) \Phi_{p}(\eta)}{(b-a)^{\alpha p + \beta-\alpha -1} f(\eta)}, $$

*where* $\eta= \max_{t \in[a, b]} u(t)$.

### Proof

By (3.1), Lemmas 3.2 and 3.3, we get

$$\begin{aligned} &u(t) \le \int_{a}^{b} G(s,s) \Phi_{q} \biggl( \int_{a}^{b} H(\tau, \tau) k(\tau)f \bigl(u(\tau) \bigr) \,\mathrm{d} \tau \biggr) \,\mathrm{d}s, \\ &\Vert u \Vert < \frac{1}{\Gamma(\alpha)} \int_{a}^{b} (b-s)^{\alpha-1} \,\mathrm{d}s \cdot \Phi_{q} \biggl( \frac{(b-a)^{\beta-1}}{4^{\beta-1} \Gamma(\beta)} \biggr) \Phi_{q} \biggl( \int_{a}^{b} k(\tau)f \bigl(u(\tau) \bigr) \, \mathrm{d} \tau \biggr) \\ &\phantom{\Vert u \Vert }= \frac{(b-a)^{\alpha}}{\Gamma(\alpha+1)} \cdot\Phi_{q} \biggl( \frac {(b-a)^{\beta-1}}{4^{\beta-1} \Gamma(\beta)} \biggr) \Phi_{q} \biggl( \int_{a}^{b} k(\tau)f \bigl(u(\tau) \bigr) \, \mathrm{d} \tau \biggr). \end{aligned}$$

Using Lemma 2.2, and taking into account that *f* is concave and nondecreasing, we see that

$$\begin{aligned} \Vert u \Vert &< \frac{(b-a)^{\alpha}}{\Gamma(\alpha+1)} \cdot\Phi _{q} \biggl( \frac{(b-a)^{\beta-1}}{4^{\beta-1} \Gamma(\beta)} \biggr) \Phi_{q} \biggl( \int_{a}^{b} k(s) \,\mathrm{d}s \biggr) \Phi_{q} \biggl( \int_{a}^{b} \frac{k(\tau)f(u(\tau)) \,\mathrm{d} \tau}{\int_{a}^{b} k(s) \,\mathrm {d}s} \biggr) \\ &< \frac{(b-a)^{\alpha}}{\Gamma(\alpha+1)} \cdot\Phi_{q} \biggl( \frac {(b-a)^{\beta-1}}{4^{\beta-1} \Gamma(\beta)} \biggr) \Phi_{q} \biggl( \int_{a}^{b} k(s) \,\mathrm{d}s \biggr) \Phi_{q} \bigl(f(\eta) \bigr), \end{aligned}$$

where $\eta= \max_{t \in[a, b]} u(t)$. Hence,

$$\int_{a}^{b} k(s) \,\mathrm{d}s > \frac{4^{\beta-1} \Gamma(\beta) \Phi_{p}(\Gamma(\alpha+1)) \Phi_{p}(\eta)}{(b-a)^{\alpha p + \beta-\alpha-1} f(\eta)}. $$

The proof is completed. □

## Applications

In the following, some applications of the obtained results are presented.

### Corollary 4.1

*If* $\lambda\in[0, 4^{\beta-1} \Gamma (\beta) \Phi_{p} (\Gamma(\alpha+1) )]$, *then the following eigenvalue problem*:

4.1 $$\begin{aligned} \textstyle\begin{cases} D_{0^{+}}^{\beta} (\Phi_{p}(^{C} D_{0^{+}}^{\alpha} y(t)) ) - \lambda \Phi_{p}(y(t))=0,\quad 0 < t < 1, \\ y^{\prime}(0)= ^{C} D_{0^{+}}^{\alpha} y(0) = 0,\qquad y(1) = ^{C} D_{0^{+}}^{\alpha} y(1) = 0, \end{cases}\displaystyle \end{aligned}$$

*has no corresponding eigenfunction* $y \in P$, *where* $1 < \alpha, \beta \leq2$, *and* $p > 1$.

### Proof

Assume that $y_{0} \in P$ is an eigenfunction of (4.1) corresponding to an eigenvalue $\lambda_{0} \in[0, 4^{\beta-1} \Gamma(\beta) \Phi_{p} (\Gamma(\alpha+1) )]$. By using Theorem 3.5 with $a=0$, $b=1$, $k(s) = \lambda_{0}$ and $M = 1$ ($f(y) = \Phi _{p}(y)$), we get

$$\lambda_{0} > 4^{\beta-1} \Gamma(\beta) \Phi_{p} \bigl(\Gamma(\alpha+1) \bigr), $$

which is a contradiction. □

From Theorems 3.4 and 3.6, we have the following.

### Corollary 4.2

*For fractional boundary value problem* (1.12), *let* $k: [a, b] \to\mathbb{R}_{+}$ *be a nontrivial Lebesgue integrable function*, *and* $f \in C(\mathbb {R}_{+}, \mathbb{R}_{+})$ *be a concave and nondecreasing function*. *If there exist two positive constants* $r_{2} > r_{1} > 0$ *such that the assumptions* (H1) *and* (H2) *hold*, *then*

$$\int_{a}^{b} k(\tau) \,\mathrm{d} \tau> \frac{4^{\beta-1} \Gamma(\beta) \Phi_{p}(\Gamma(\alpha+1)) \Phi_{p}(r_{1})}{(b-a)^{\alpha p + \beta-\alpha-1} f(r_{2})}. $$

### Example 4.3

Consider the following fractional boundary value problem:

$$\textstyle\begin{cases} D_{0^{+}}^{3/2} (\Phi_{1.8}(^{C} D_{0^{+}}^{4/3} y) ) - \sqrt{t} \ln(15+y)=0,\quad 0 < t < 1, \\ y^{\prime}(0)= ^{C} D_{0^{+}}^{4/3} y(0) = 0,\qquad y(1) = ^{C} D_{0^{+}}^{4/3} y(1) = 0. \end{cases} $$

Obviously, we have

(i) $f(y) = \ln(15+y): \mathbb{R}_{+} \to\mathbb{R}_{+}$ is continuous, concave and nondecreasing;
(ii) $k(t) = \sqrt{t}: [0, 1] \to\mathbb{R}_{+}$ is a Lebesgue integrable function with $\int_{0}^{1} k(t) \,\mathrm{d}t = \frac{2}{3} > 0$.

We now compute the values of *ρ* and *ω* in (H1) and (H2), respectively.

Since $A_{3/2} = ( (\frac{3}{4} )^{1/2} - (\frac{1}{4} )^{1/2} )^{2} = 1 - \frac{\sqrt{3}}{2}$, we have $c_{3/2} = \frac{\frac{3}{4} - A_{3/2}}{1 - A_{3/2}} = 1- \frac {\sqrt{3}}{6}$. where $A_{3/2}$ and $c_{3/2}$ ($\beta= 3/2$) are as in (3.8). Hence

$$\sigma(s) = \textstyle\begin{cases} \frac{\sqrt{3}}{2} \frac{(1-s)^{1/2} - 1}{(s (1-s))^{1/2}} & \text{if } s \in (0, 1- \frac{\sqrt{3}}{6} ], \\ \frac{1}{2 s^{1/2}} & \text{if } s \in [ 1- \frac{\sqrt{3}}{6}, 1 ). \end{cases} $$

Thus, by a simple computation, we obtain

$$\rho\approx61.7797,\qquad \omega\approx3.8213. $$

Choosing $r_{1} = 1/50 $ and $r_{2} = 1$, we obtain

1. $f(y) = \ln(15+y) \ge\rho\Phi_{1.8}(r_{1})$ for $y \in[0, 1/50]$;
2. $f(y) = \ln(15+y) \le\omega\Phi_{1.8}(r_{2})$ for $y \in[0, 1]$.

Hence, from Corollary 4.2, we obtain

$$\int_{0}^{1} k(t) \,\mathrm{d}t > \frac{2 \Gamma(3/2) \Phi_{1.8} (\frac{1}{50}\Gamma (7/3) )}{\ln16} \approx 0.0321. $$

## Conclusions

In this paper, we prove existence of positive solutions to a nonlinear fractional boundary value problem involving a *p*-Laplacian operator. Then, under some mild assumptions on the nonlinear term, we present two new Lyapunov-type inequalities. A numerical example shows that the new results are efficient.

## References

[1] Lyapunov AM (1907). Problème général de la stabilité du mouvement. Ann. Fac. Sci. Univ. Toulouse.

[2] Brown RC, Hinton DB, Rassias TM (2000). Lyapunov inequalities and their applications. Survey on Classical Inequalities.

[3] Tiryaki A (2010). Recent developments of Lyapunov-type inequalities. Adv. Dyn. Syst. Appl..

[4] Ferreira RAC (2014). On a Lyapunov-type inequality and the zeros of a certain Mittag-Leffler function. J. Math. Anal. Appl..

[5] Ma D (2016). A generalized Lyapunov inequality for a higher-order fractional boundary value problem. J. Inequal. Appl..

[6] Dhar S, Kong Q, McCabe M (2016). Fractional boundary value problems and Lyapunov-type inequalities with fractional integral boundary conditions. Electron. J. Qual. Theory Differ. Equ..

[7] Ferreira RAC (2013). A Lyapunov-type inequality for a fractional boundary value problem. Fract. Calc. Appl. Anal..

[8] Jleli M, Samet B (2015). Lypunov-type inequalities for a fractional differential equation with mixed boundary conditions. Math. Inequal. Appl..

[9] Jleli M, Kirane M, Samet B (2017). Lyapunov-type inequalities for fractional partial differential equations. Appl. Math. Lett..

[10] Jleli M, Nieto JJ, Samet B (2017). Lyapunov-type inequalities for a higher order fractional differential equation with fractional integral boundary conditions. Electron. J. Qual. Theory Differ. Equ..

[11] Rong J, Bai C (2015). Lyapunov-type inequality for a fractional differential equation with fractional boundary conditions. Adv. Differ. Equ..

[12] Arifi NA, Altun I, Jleli M, Lashin A, Samet B (2016). Lyapunov-type inequalities for a fractional *p*-Laplacian equation. J. Inequal. Appl..

[13] Chidouh A, Torres DFM (2017). A generalized Lyapunov’s inequality for a fractional boundary value problem. J. Comput. Appl. Math..

[14] Miller KS, Ross B (1993). An Introduction to the Fractional Calculus and Fractional Differential Equations.

[15] Podlubny I (1999). Fractional Differential Equations.

[16] Guo G, Lakshmikantham V (1988). Nonlinear Problems in Abstract Cones.

[17] Rudin W (1987). Real and Complex Analysis.

